# Envenoming by *Viridovipera stejnegeri* snake: a patient with liver cirrhosis presenting disruption of hemostatic balance

**DOI:** 10.1186/s40409-017-0096-9

**Published:** 2017-02-14

**Authors:** Chih-Ying Chien, Shu-Chen Liao, Chien-Hung Liao, Ting-Shuo Huang, Yu-Hsien Chen

**Affiliations:** 1 Department of General Surgery, Chang-Gung Memorial Hospital, Keelung Branch, 222, Maijin Road, Keelung, Taiwan; 2 Department of Emergency Medicine, Chang Gung Memorial Hospital, Keelung, Taiwan; 3 Department of Trauma and Emergency Surgery, Chang Gung Memorial Hospital, Linkou, Taiwan

**Keywords:** Snakebite, Liver cirrhosis, Coagulopathy

## Abstract

**Background:**

In most cases of envenoming by the green habu *Viridovipera stejnegeri* in Taiwan coagulopathy is not observed.

**Case presentation:**

Herein, we describe the case of a patient with liver cirrhosis who developed venom-induced consumptive coagulopathy after *V. stejnegeri* bite. Laboratory investigation revealed the following: prothrombin time > 100 s (international normalized ratio > 10), activated partial thromboplastin time > 100 s, fibrinogen < 50 mg/dL, and fibrin degradation product > 80 μg/mL. The patient recovered after administration of bivalent hemorrhagic antivenom, vitamin K, fresh frozen plasma and cryoprecipitate.

**Conclusion:**

The liver, directly involved in the acute phase reaction, is the main responsible for neutralization of animal toxins. Any patient with history of liver cirrhosis bitten by a venomous snake, even those whose venoms present low risk of coagulopathy, should be very carefully monitored for venom-induced consumptive coagulopathy (VICC), since the hemostatic balance may be disrupted.

## Background

Six species of venomous snakes are commonly found in Taiwan. Most cases of snake envenoming admitted to hospital emergency departments (ED) are caused by *Protobothrops mucrosquamatus* (Taiwan habu) and *Viridovipera stejnegeri* (green habu, bamboo viper or Chinese green tree viper) [1]. The latter is very easily distinguishable from other species because of its markedly different color pattern. Unlike other species that are brown, black or white, *V. stejnegeri* has a unique color characteristic, its dorsal scales are bright to dark green whereas its ventral scales are white to pale green and its tail is reddish. *V. stejnegeri* bites provoke less severe clinical effects than the other local species. For example, systemic coagulopathy is extremely rare after *V. stejnegeri* envenoming [2]. Venom-induced consumptive coagulopathy (VICC) has been introduced for a more general description of this type of systemic coagulopathy [3]. Herein, we present a rare case of a patient with liver cirrhosis who developed VICC after *V. stejnegeri* envenoming.

## Case presentation

**Fig. 1:**
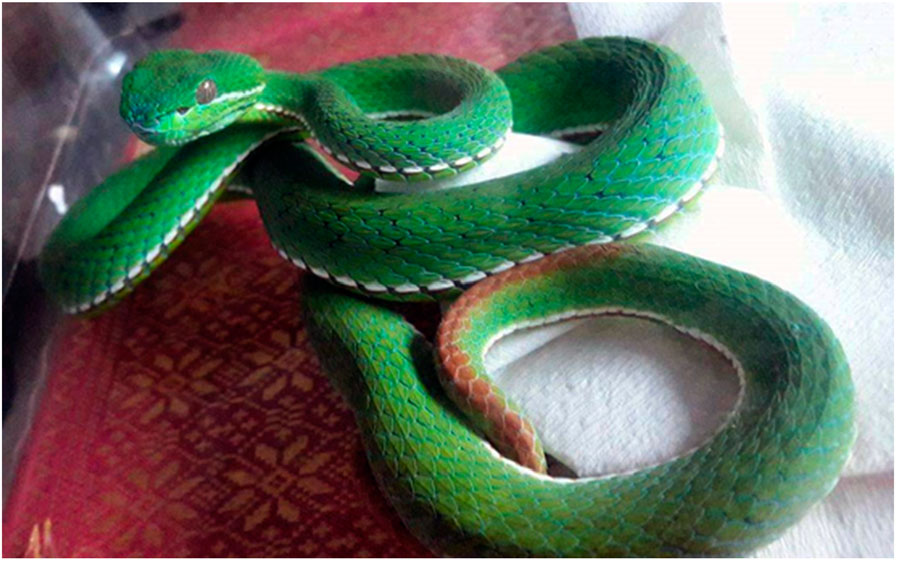
The color pattern of *Viridovipera stejnegeri* (green habu) is bright to dark green on the dorsal side and pale green to whitish on the ventral side

A 47-year-old man was bitten by a venomous snake on his right index finger at his home, in a mountainous area, when he was trying to get rid of the animal. One hour after the bite, he was sent to a local hospital for first aid. The staff of the hospital showed pictures of snakes to the patient, who identified the snake that had bitten him as *Viridovipera stejnegeri* (Fig. 1).

Then, the man received four vials of bivalent hemorrhagic antivenom. Because of pain and swelling on the right hand and elbow, he was transferred to our ED for further treatment. Initial clinical examination revealed stable vital signs with a normal level of consciousness. No dyspnea or muscle weakness was observed. However, swelling and tenderness were observed on his right index finger and elbow. A fang mark was observed on the right index finger. On arrival to the ED, laboratory investigation revealed a platelet count of 142,000/μL, prothrombin time (PT) >100 s (international normalized ratio > 10), and activated partial thromboplastin time (APTT) > 100 s (Table 1). Both creatinine and bilirubin levels were normal. We found that he had been a hepatitis C virus carrier for several years. He also had liver cirrhosis, Child–Pugh score A and diabetes mellitus. A surgical history of splenectomy and left nephrectomy caused by a trauma accident 10 years ago was also noted. He was under outpatient follow up of the gastroenterology department without coagulopathy noted before this episode of snake envenoming. Two vials of bivalent hemorrhagic antivenom were given. Blood transfusion with two units of fresh frozen plasma (FFP) and one vial of vitamin K (10 mg) were administered. Fluid replacement therapy with isotonic normal saline (0.9%) was also used for treatment.

**Table 1:** Time course of envenoming (biochemical parameters) since the bite until hospital discharge

	PT (s)	INR	APTT (s)	Plt 1000/μL	Fibrinogen (mg/dL)	FDP (μg/mL)	D-dimer FEUmg/L
Day 1	>100	>10	>100	142			
Day 2	29.8	2.71	29.4				
Day 3	>100	>10					
Day 4	>100	>10	29	159			
Day 5	>100	>10	28.3		<50	>80	1.63
Day 6	12	1.15	26.4	172	65	>80	
Day 7	12.2	1.17			80	>80	
Day 9	11.4	1.1			122	>80	
Day 14	9.7	0.95		401	284	<10	

*PT* prothrombin time, *INR* international normalized ratio, *Plt* platelet, *APTT* activated partial thromboplastin time, *FDP* fibrin degradation product

The patient was admitted to the general ward and continued to receive antivenom, vitamin K and FFP until the PT level improved. On day 5, the fibrinogen level was low at < 50 mg/dL, and the fibrin degradation product (FDP) was > 80 μg/mL. Cryoprecipitate was given for several days until the fibrinogen level improved. The local toxic effect on the right hand improved. He was discharged on day 9. The man returned to our outpatient department 2 weeks after the snakebite. The laboratory data revealed that PT, APTT, fibrinogen and FDP were all within the normal range. He returned to work 1 week later. He was under outpatient department follow up and the laboratory data of PT, APTT, albumin and bilirubin level were all within normal range 1 year later.

## Discussion

Approximately 40 cases of snakebite are admitted to Chang-Gung Memorial Hospital, Keelung branch in Taiwan every year. Most events are observed during summer and fall, whereas the areas where they are mostly like to occur are farms, gardens or yards in mountainous regions. When a snakebite patient is sent to our ED, the first step is to confirm the snake species. Subsequently, we will give the appropriate antivenom depending on the species. In Taiwan, the available antivenom is equine F (ab′) 2 manufactured by the Centers for Disease Control (CDC) of Taiwan [4].

Most cases of envenoming referred to our hospital are caused by *P. mucrosquamatus* and *V. stejnegeri* snakes. *P. mucrosquamatus* envenoming is associated with more serious local and systemic complications, such as VICC, acute renal failure and rhabdomyolysis, in comparison with *V. stejnegeri* envenoming [2]. The toxins of *P. mucrosquamatus* and *V. stejnegeri* venoms contain phospholipases A_2_ (PLA_2_), metalloproteinases, serine proteinases and C-type lectin-like related proteins [5]. PLA_2_ is an anticoagulant enzyme that can cause extrinsic coagulation pathway inhibition by inhibiting extrinsic tenase complex and prothrombinase complex [6, 7]. Metalloproteinases are fibrinongenases that can destroy fibrinogen and serine proteinases, such as protein C activators, can inhibit coagulation. C-type lectin-like related proteins can inhibit prothrombinase activation, causing anticoagulation [8]. *P. mucrosquamatus* venom has a much stronger anticoagulant effect than that of *V. stejnegeri* [9]. In Taiwan, *V. stejnegeri* envenoming rarely causes VICC [2]. Only one study reported that *V. stejnegeri* envenoming may have caused a disseminated intravascular coagulation-like syndrome in south China [10].

In our case, the patient was a hepatitis C virus carrier, consumed alcohol and had liver cirrhosis, Child–Pugh score A. He developed VICC after *V. stejnegeri* envenoming. The liver, directly involved in the acute phase reaction, is the main organ that neutralizes animal toxins [11–14]. It is the predominant site for producing the coagulation factors including fibrinogen (factor I), prothrombin (factor II), upstream factors V, VII, IX, X and XI and anticoagulant proteins. The liver produces some inhibitors of coagulation, including protein S, protein C, antithrombin and fibrinolytic factors. Stable liver cirrhosis may maintain rebalanced hemostasis because both procoagulant factors and fibrinolytic proteins are deficient [15]. An unstable hemostatic balance can be disrupted by acute phase reaction, such as infection and inflammation, and oxidative stress induced by the venom [12, 13, 16, 17].

In our patient, highly elevated PT (>100 s) and APTT (>100 s) levels were detected, possibly because of snake envenoming and liver coagulation factor deficiency. We suspected that *V. stejnegeri* envenoming disrupted the rebalanced hemostasis. He initially had mild thrombocytopenia (142,000/μL), which gradually improved, possibly because of his history of splenectomy, which may have been responsible for splenic sequestration of platelets. We gave him bivalent hemorrhagic antivenom to neutralize the toxins and FFP and vitamin K to correct coagulopathy. His fibrinogen level was low (<50 mg/dL) on day 5, therefore cryoprecipitate transfusions were necessary. Edema and swelling were initially observed in his local wound, but gradually improved. He easily developed skin ecchymosis at the intravenous injection site.

Only a few studies have discussed *V. stejnegeri* envenoming in patients with liver cirrhosis. VICC is extremely rare in patients bitten by *V. stejnegeri*. Balanced hemostasis in patients with liver cirrhosis who are attacked by venomous snakes may be disrupted, and these patients should be very carefully monitored to avoid catastrophic hemorrhage complications. The use of clotting factors such as FFP or cryoprecipitate to treat VICC without bleeding is still controversial [18]. However, patients with liver cirrhosis cannot resynthesize coagulation factors as rapidly as healthy ones. Early replacement of clotting factors after antivenom treatment can ensure early recovery of clotting function [19].

## Conclusions

*V. stejnegeri* envenoming is associated with a relatively low risk of VICC in Taiwan. However, any patient with a history of liver cirrhosis who is bitten by a venomous snake should be very carefully monitored for VICC. The likelihood of VICC may be higher in such patients. We must evaluate closely the laboratory data and symptoms. Such patients should be observed for 24 h, and laboratory data should be followed up to determine whether VICC has occurred. In case of VICC, patients should be admitted for treatment until improvement of clotting function.

## Abbreviations

APTT: Activated partial thromboplastin time
CDC: Centers for Disease Control
DIC: Disseminated intravascular coagulation
ED: Emergency department
FDP: Fibrin degradation product
FFP: Fresh frozen plasma
INR: International normalized ratio
PLA_2_: Phospholipase A_2_
Plt: Platelet
PT: Prothrombin time
VICC: Venom-induced consumptive coagulopathy

## References

[1] Hung DZ (2004). Taiwan’s venomous snakebite: epidemiological, evolution and geographic differences. Trans R Soc Trop Med Hyg.

[2] Chen YW, Chen MH, Chen YC, Hung DZ, Chen CK, Yen DH (2009). Differences in clinical profiles of patients with *Protobothrops mucrosquamatus* and *Viridovipera stejnegeri* envenoming in Taiwan. Am J Trop Med Hyg.

[3] Isbister GK (2009). Procoagulant snake toxins: laboratory studies, diagnosis, and understanding snakebite coagulopathy. Semin Thromb Hemost.

[4] Chen JC, Liaw SJ, Bullard MJ, Chiu TF (2000). Treatment of poisonous snakebites in northern Taiwan. J Formos Med Assoc.

[5] Villalta M, Pla D, Yang SL, Sanz L, Segura A, Vargas M (2012). Snake venomics and antivenomics of *Protobothrops mucrosquamatus* and *Viridovipera stejnegeri* from Taiwan: keys to understand the variable immune response in horses. J Proteomics.

[6] Kini RM, Evans HJ (1987). Structure-function relationships of phospholipases. The anticoagulant region of phospholipases A2. J Biol Chem.

[7] Stefansson S, Kini RM, Evans HJ (1989). The inhibition of clotting complexes of the extrinsic coagulation cascade by the phospholipase A2 isoenzymes from *Naja nigricollis* venom. Thromb Res.

[8] Ouyang C, Yang FY (1975). Purification and properties of the anticoagulant principle of *Trimeresurus gramineus* venom. Biochim Biophys Acta.

[9] Liao WB, Lee CW, Tsai YS, Liu BM, Chung KJ (2000). Influential factors affecting prognosis of snakebite patients management: Kaohsiung Chang Gung Memorial Hospital experience. Chang Gung Med J.

[10] Li QB, Huang GW, Kinjoh K, Nakamura M, Kosugi T (2001). Hematological studies on DIC-like findings observed in patients with snakebite in south China. Toxicon.

[11] Barraviera B, Bonjorno Junior JC, Arkaki D, Domingues MA, Pereira PC, Mendes RP (1989). A retrospective study of 40 victims of *crotalus* snake bites. Analysis of the hepatic necrosis observed in one patient. Rev Soc Bras Med Trop.

[12] Barraviera B, Lomonte B, Tarkowski A, Hanson LÅ, Meira DA (1995). Acute-phase reactions, including cytokines, in patients bitten by *Bothrops* and *Crotalus* snakes in brazil. J Venom Anim Toxins.

[13] Barraviera B, Coelho KY, Curi PR, Meira DA (1995). Liver dysfunction in patients bitten by *Crotalus Durissus Terrificus* (Laurenti, 1768) snakes in Botucatu (State of Sao Paulo, Brazil). Rev Inst Med Trop Sao Paulo.

[14] França RF, Vieira RP, Ferrari EF, Souza RA, Osorio RAL, Prianti-Jr ACG (2009). Acute hepatotoxicity of *Crotalus durissus terrificus* (South American rattlesnake) venom in rats. J Venom Anim Toxins incl Trop Dis.

[15] Lisman T, Porte RJ (2010). Rebalanced hemostasis in patients with liver disease: evidence and clinical consequences. Blood.

[16] Hou MC, Lin HC, Liu TT, Kuo BI, Lee FY, Chang FY (2004). Antibiotic prophylaxis after endoscopic therapy prevents rebleeding in acute variceal hemorrhage: a randomized trial. Hepatology.

[17] Al-Quraishy S, Dkhil MA, Abdel Moneim AE (2014). Hepatotoxicity and oxidative stress induced by *Naja haje* crude venom. J Venom Anim Toxins incl Trop Dis.

[18] Maduwage K, Isbister GK (2014). Current treatment for venom-induced consumption coagulopathy resulting from snakebite. PLoS Negl Trop Dis.

[19] Brown SG, Caruso N, Borland ML, McCoubrie DL, Celenza A, Isbister GK (2009). Clotting factor replacement and recovery from snake venom-induced consumptive coagulopathy. Intensive Care Med.

